# Efficacy and safety of follitropin alfa/lutropin alfa in ART: a randomized controlled trial in poor ovarian responders

**DOI:** 10.1093/humrep/dew360

**Published:** 2017-01-28

**Authors:** P. Humaidan, W. Chin, D. Rogoff, T. D'Hooghe, S. Longobardi, J. Hubbard, J. Schertz, on behalf of the ESPART Study Investigators

**Affiliations:** 1 The Fertility Clinic, Skive Regional Hospital, Skive, Denmark; 2 Faculty of Health, Aarhus University, Aarhus, Denmark; 3 Global Biostatistics and Epidemiology, EMD Serono, Billerica, MA, USA, a business of Merck KGaA, Darmstadt, Germany; 4 Global Clinical Development, EMD Serono Research and Development Institute, Billerica, MA, USA, a business of Merck KGaA, Darmstadt, Germany; 5 Global Medical Affairs Fertility, Merck KGaA, Darmstadt, Germany

**Keywords:** POR, poor ovarian response, follitropin alfa, lutropin alfa, FSH, LH, Bologna criteria, IVF, ICSI, ART

## Abstract

**STUDY QUESTION:**

How does the efficacy and safety of a fixed-ratio combination of recombinant human FSH plus recombinant human LH (follitropin alfa plus lutropin alfa; r-hFSH/r-hLH) compare with that of r-hFSH monotherapy for controlled ovarian stimulation (COS) in patients with poor ovarian response (POR)?

**SUMMARY ANSWER:**

The primary and secondary efficacy endpoints were comparable between treatment groups and the safety profile of both treatment regimens was favourable.

**WHAT IS KNOWN ALREADY:**

Although meta-analyses of clinical trials have suggested some beneficial effect on reproductive outcomes with r-hLH supplementation in patients with POR, the definitions of POR were heterogeneous and limit the comparability across studies.

**STUDY DESIGN, SIZE, DURATION:**

Phase III, single-blind, active-comparator, randomized, parallel-group clinical trial. Patients were followed for a single ART cycle. A total of 939 women were randomized (1:1) to receive either r-hFSH/r-hLH or r-hFSH. Randomization, stratified by study site and participant age, was conducted* via* an interactive voice response system.

**PARTICIPANTS/MATERIALS, SETTING, METHODS:**

Women classified as having POR, based on criteria incorporating the ESHRE Bologna criteria, were down-regulated with a long GnRH agonist protocol and following successful down-regulation were randomized (1:1) to COS with r-hFSH/r-hLH or r-hFSH alone. The primary efficacy endpoint was the number of oocytes retrieved following COS. Safety endpoints included the incidence of adverse events, including ovarian hyperstimulation syndrome (OHSS). *Post hoc* analyses investigated safety outcomes and correlations between live birth and baseline characteristics (age and number of oocytes retrieved in previous ART treatment cycles or serum anti-Müllerian hormone (AMH)). The significance of the treatment effect was tested by generalized linear models (Poisson regression for counts and logistic regression for binary endpoints) adjusting for age and country.

**MAIN RESULTS AND THE ROLE OF CHANCE:**

Of 949 subjects achieving down-regulation, 939 were randomized to r-hFSH/r-hLH (*n* = 477) or r-hFSH (*n* = 462) and received treatment.

Efficacy assessment: In the intention-to-treat (ITT) population, the mean (SD) number of oocytes retrieved (primary endpoint) was 3.3 (2.71) in the r-hFSH/r-hLH group compared with 3.6 (2.82) in the r-hFSH group (between-group difference not statistically significant). The observed difference between treatment groups (r-hFSH/r-hLH and r-hFSH, respectively) for efficacy outcomes decreased over the course of pregnancy (biochemical pregnancy rate: 17.3% versus 23.9%; clinical pregnancy rate: 14.1% versus 16.8%; ongoing pregnancy rate: 11.0% versus 12.4%; and live birth rate: 10.6% versus 11.7%). An interaction (identified *post hoc*) between baseline characteristics related to POR and treatment effect was noted for live birth, with r-hFSH/r-hLH associated with a higher live birth rate for patients with moderate or severe POR, whereas r-hFSH was associated with a higher live birth rate for those with mild POR. A *post hoc* logistic regression analysis indicated that the incidence of total pregnancy outcome failure was lower in the r-hFSH/r-hLH group (6.7%) compared with the r-hFSH group (12.4%) with an odds ratio of 0.52 (95% CI 0.33, 0.82; *P* = 0.005).

Safety assessment: The overall proportion of patients with treatment-emergent adverse events (TEAEs) occurring during or after r-hFSH/r-hLH or r-hFSH use (stimulation or post-stimulation phase) was 19.9% and 26.8%, respectively. There was no consistent pattern of TEAEs associated with either treatment.

**LIMITATIONS, REASONS FOR CAUTION:**

Despite using inclusion criteria for POR incorporating the ESHRE Bologna criteria, further investigation is needed to determine the impact of the heterogeneity of POR in the Bologna patient population. The observed correlation between baseline clinical characteristics related to POR and live birth rate, as well as the observed differences between groups regarding total pregnancy outcome failure were from *post hoc* analyses, and the study was not powered for these endpoints. In addition, the attrition rate for pregnancy outcomes in this trial may not reflect general medical practice. Furthermore, as the patient population was predominantly White these results might not be applicable to other ethnicities.

**WIDER IMPLICATIONS OF THE FINDINGS:**

In the population of women with POR investigated in this study, although the number of oocytes retrieved was similar following stimulation with either a fixed-ratio combination of r-hFSH/r-hLH or r-hFSH monotherapy, *post hoc* analyses showed that there was a lower rate of total pregnancy outcome failure in patients receiving r-hFSH/r-hLH, in addition to a higher live birth rate in patients with moderate and severe POR. These findings are clinically relevant and require additional investigation. The benefit:risk balance of treatment with either r-hFSH/r-hLH or r-hFSH remains positive.

**STUDY FUNDING/COMPETING INTEREST(S):**

This study was funded by Merck KGaA, Darmstadt, Germany. P.H. has received honoraria for lectures and unrestricted research grants from Ferring, Merck KGaA and MSD. D.R. is a former employee of EMD Serono, a business of Merck KGaA, Darmstadt, Germany. J.S., J.H. and W.C. are employees of EMD Serono Research and Development Institute, a business of Merck KGaA, Darmstadt, Germany. T.D.’H. and S.L. are employees of Merck KGaA, Darmstadt, Germany.

**TRIAL REGISTRATION NUMBER:**

ClinicalTrials.gov identifier: NCT02047227; EudraCT Number: 2013-003817-16.

**TRIAL REGISTRATION DATE:**

ClinicalTrials.gov: 24 January 2014; EudraCT: 19 December 2013.

**DATE OF FIRST PATIENT'S ENROLMENT:**

30 January 2014.

## Introduction

One in six couples worldwide will experience at least one infertility problem during their reproductive years (ESHRE, 2014) and the majority will benefit from ART. Between 5.6% and 35.1% of women will exhibit poor ovarian response (POR) (Oudendijk *et al*., 2012). POR does not have a single cause and the population with POR is, therefore, heterogeneous and difficult to concisely characterize, with clinical trials conducted to date using diverse defining criteria (Ferraretti *et al*., 2011; Polyzos and Devroey, 2011; Polyzos and Sunkara, 2015; Papathanasiou *et al*., 2016). The most consistent variable affecting ovarian response is age and POR is associated with chronological ageing (Ferraretti *et al*., 2011), with subfertility becoming more pronounced after the age of 35 years (The ESHRE Capri Workshop Group, 2010). In addition, an age-related decline in response to exogenous gonadotropin stimulation and a reduction in the number of oocytes, oocyte quality, fertilization rate, number of embryos, implantation rate and, ultimately, live birth rate have been well documented (Nelson *et al*., 2013). Owing to social changes, more women delay childbearing and this has resulted in a greater number of women in their late thirties and early forties seeking infertility treatment, highlighting the need for studies in patients with POR (The ESHRE Capri Workshop Group, 2010; Kocourkova *et al*., 2014).

A systematic review identified 47 randomized controlled trials that used 41 different descriptions for POR, with each definition used by no more than three trials (Polyzos and Devroey, 2011). This disparity in the definitions used makes it challenging to compare between, and draw conclusions from, clinical trials investigating women with POR and has meant that, in general, the results of studies and meta-analyses should be interpreted with caution when considering their applicability to clinical practice. To address this lack of consistency, the ESHRE Bologna criteria were developed by consensus in 2011 as an attempt to standardize the definition of POR for use in clinical trials, and to better enable their comparison (Ferraretti *et al*., 2011).

However, following the development of these criteria, no adequately powered, prospective, randomized controlled trials have investigated treatment in this population, and an ideal protocol for controlled ovarian stimulation (COS) in women with POR has yet to be identified. There is, however, some evidence that supplementing recombinant human FSH (r-hFSH) with recombinant human LH (r-hLH) during ART may have beneficial effects on outcomes in women with POR (Bosch *et al*., 2011; Hill *et al*., 2012; Lehert *et al*., 2014). Supplementation with LH may be beneficial owing to increased FSH receptor expression and growth, in addition to improved follicular recruitment and a reduced rate of granulosa cell apoptosis (Hillier, 2001; Ruvolo *et al*., 2007).

A meta-analysis by Lehert *et al*. (2014), which included 6443 patients from 40 randomized controlled trials (45 quantitative studies in total), found that there was no difference in the number of oocytes retrieved from women in the overall analysis population who were treated with r-hFSH/r-hLH compared with those treated with r-hFSH alone. However, when women with POR (*n* = 1077) were considered separately, significantly more oocytes were retrieved with r-hFSH/r-hLH compared with r-hFSH (weighted mean difference +0.75 oocytes; 95% CI 0.14, 1.36) (Lehert *et al*., 2014). In addition, significantly higher clinical pregnancy rates were observed with r-hFSH/r-hLH compared with r-hFSH in both the overall population (risk ratio [RR] 1.09; 95% CI 1.01, 1.18) and the subpopulation of women with POR (RR 1.30; 95% CI 1.01, 1.67), with a greater observed difference between treatments observed in the POR group (Lehert *et al*., 2014). However, the definitions of POR were heterogeneous among these studies because the criteria were defined by the authors of each study included in the meta-analysis, as all studies preceded the publication of the Bologna criteria. Nonetheless, these study data represented the best available at the time.

The Efficacy and Safety of Pergoveris in Assisted Reproductive Technology (ESPART) trial was designed to investigate the hypothesis that a fixed-ratio (2:1) combination of r-hFSH/r-hLH was generally safe and superior to r-hFSH alone, in terms of the number of oocytes retrieved, for COS in patients with POR. The POR inclusion criteria used in this trial incorporated the Bologna criteria (Table I). Fixed-ratio (2:1) combination follitropin alfa/lutropin alfa (r-hFSH 150 IU plus r-hLH 75 IU; Pergoveris^®^; Merck KGaA, Darmstadt, Germany) is indicated for stimulation of follicular development in women with severe LH and FSH deficiency, defined by an endogenous serum LH level <1.2 IU/l. Additionally in some countries, it is indicated for COS in sub-optimal responders, defined as having a previous response to COS characterized by either a small number (<7) of pre-ovulatory follicles or the use of high FSH doses (≥3000 IU per cycle) or advanced (≥35 years) maternal age. Follitropin alfa (GONAL-f^®^; Merck KGaA) is indicated for anovulation in women who have been unresponsive to treatment with clomiphene citrate and for stimulation of multifollicular development in women undergoing superovulation for ART and in association with an LH preparation for stimulation of follicular development in women with severe LH and FSH deficiency.

**Table I:** The ESHRE Bologna criteria and the ESPART trial inclusion criteria for POR.

2011 ESHRE Bologna criteria, Ferraretti *et al*. (2011)	ESPART POR inclusion criteria*
Advanced maternal age (≥40 years) or any other risk factor	Advanced maternal age (≥40–<41 years, i.e. patients between their 40th and 41st birthday)
A previous POR (cycles cancelled or ≤3 oocytes with a conventional protocol)	Previous ART cycle with ≤3 oocytes retrieved with a conventional stimulation protocol
An abnormal ORT (AFC <5–7 follicles or AMH <0.5–1.1 ng/ml)	An abnormal ORT (AMH 0.12–1.3 ng/ml; measured by AMH GEN II ELISA, Beckman Coulter, Inc., High Wycombe, UK) La Marca and Sunkara (2014)
In the absence of advanced maternal age or abnormal ORT, two previous episodes of POR after maximal stimulation	Patients with two previous episodes of POR after maximal stimulation were excluded

^*^Two out of three POR inclusion criteria needed to be met for inclusion in the ESPART trial.

AFC, antral follicle count; ORT, ovarian reserve test; POR, poor ovarian response; ESPART, Efficacy and Safety of Pergoveris in Assisted Reproductive Technology.

## Materials and Methods

ESPART was a Phase III, randomized, single-blind, parallel-group, active-comparator clinical trial in women undergoing IVF and/or ICSI (ClinicalTrials.gov identifier: NCT02047227; EudraCT Number: 2013-003817-16). A detailed description of the methodology has been published previously (Humaidan *et al*., 2015) and is summarized below. The study was performed in accordance with the clinical trial protocol, the ethical principles that have their origin in the Declaration of Helsinki, the International Conference on Harmonization–Good Clinical Practice guidelines and all applicable regulatory requirements. All participants provided written informed consent prior to entry.

### Study participants

Women were included in ESPART if they were ≥18–<41 years old, had a BMI between 18 and 31 kg/m^2^ (inclusive), were eligible for COS and ART treatment (including ICSI) and had a diagnosis of POR based upon criteria incorporating the 2011 ESHRE Bologna criteria (Table I) (Ferraretti *et al*., 2011). At least two of the POR inclusion criteria had to be met for inclusion in ESPART. The ESHRE Bologna criteria were not utilized as published, rather a stricter interpretation was applied to remove diagnostic subjectivity, reduce patient heterogeneity and exclude patients with the worst reproductive prognosis. In order to achieve this, the trial excluded women with other ‘risk factors for POR’, women aged ≥41 years, and women with at least two previous episodes of POR after maximal stimulation. Furthermore, the upper threshold of serum AMH was increased to 1.3 ng/ml based on evidence that this cutoff level is superior to 1.1 ng/ml for POR prediction (Ferraretti and Gianaroli, 2014; La Marca and Sunkara, 2014). Additional key exclusion criteria included primary ovarian failure and the use of preimplantation genetic screening or diagnosis.

### Study treatments and interventions

The trial duration for each patient was a maximum of 365 days, and the trial was conducted between January 2014 and February 2015. Following pituitary down-regulation with daily triptorelin acetate (Decapeptyl^®^; Ferring Pharmaceuticals, Saint-Prex, Switzerland) and a negative pregnancy test, women were randomized 1:1 to undergo COS with either a fixed-ratio combination of r-hFSH 300 IU plus r-hLH 150 IU (follitropin alfa/lutropin alfa; Pergoveris^®^) or r-hFSH 300 IU monotherapy (follitropin alfa; GONAL-f^®^), with the dose fixed for the first 4 days of COS. Women were randomized *via* an interactive voice response system (Cenduit GmbH, Switzerland), and randomization was stratified by study site and participant age (<35 or ≥35 years).

The investigator and all site personnel (with the exception of the trial nurse/coordinator and/or pharmacist/pharmacy assistant who informed participants of their treatment) were blinded to treatment allocation throughout the duration of the trial. A special agreement for respecting the blinding procedure was signed by all personnel involved in the trial, and each site documented the procedures used to maintain the blind. In the case of dosage adjustment, the blind was kept intact with generic instructions given to the trial nurse or pharmacist by the investigator, for both trial drugs. The biostatistics team remained blinded to the treatment codes until the database was locked, the Statistical Analysis Plan finalized and the analysis sets agreed. This single-blind design was implemented as the quality and scientific value of the data obtained from an assessor-blind design ensures the highest level of integrity while reducing patients’ treatment burdens resulting from the implementation of a double blind, double-dummy design.

A long GnRH agonist protocol was used to ensure that the contribution of endogenous LH was minimized, to allow for a fair assessment of the exogenous LH being administered in the group receiving r-hFSH/r-hLH. Triptorelin acetate 0.1 mg was administered daily from cycle day 20–21 after confirmed ovulation in subjects with spontaneous menses or from cycle day 3–4 in anovulatory or oligo-ovulatory subjects with induced menses. Pituitary down-regulation, confirmed by a serum oestradiol (E_2_) level ≤50 pg/ml, was assessed after 14 days of triptorelin acetate treatment. If down-regulation was not confirmed, treatment with triptorelin acetate continued for an additional 7 days. If E_2_ was > 50 pg/ml at Day 21, the subject was excluded from further treatment in the trial. Triptorelin acetate administration was continued in subjects with confirmation of down-regulation until the administration of recombinant hCG (r-hCG; Ovidrel^®^/Ovitrelle^®^ Prefilled Syringe, Merck KGaA) to trigger final follicular maturation.

Within 4 days after confirmation of down-regulation, either r-hFSH/r-hLH or r-hFSH was administered concurrently with daily triptorelin acetate. Dose adjustments of r-hFSH (either increases or decreases in 75 IU increments, with concomitant automatic adjustment of r-hLH in participants treated with r-hFSH/r-hLH owing to the combined formulation) were allowed after the first 4 days of stimulation, as monitored by study investigators. The maximum allowed daily dose was 450 IU r-hFSH (plus 225 IU r-hLH in the r-hFSH/r-hLH group).

Once follicle(s) reached a mean diameter of 17–18 mm, a single injection of r-hCG 250 μg was administered to trigger final follicular maturation. Oocyte retrieval was performed 34–38 h after r-hCG administration, and embryo transfer took place according to each centre's standard practice (maximum three embryos), 2–3 days after oocyte retrieval.

### Study objectives and efficacy endpoints

The primary objective of the ESPART study was to demonstrate superiority of a fixed-ratio combination of r-hFSH/r-hLH compared with r-hFSH in women with POR undergoing COS for IVF/ICSI. The primary efficacy endpoint was the total number of oocytes retrieved per participant. Secondary endpoints included biochemical pregnancy defined as a positive serum hCG determination 15–20 days after r-hCG injection; clinical pregnancy defined as the presence of at least one ultrasound-confirmed gestational sac in the uterus with or without foetal heart activity 35–42 days after r-hCG injection; ongoing pregnancy rate defined as the presence of at least one viable foetus with positive heart activity 10 weeks after embryo transfer; live birth rate; and implantation rate defined as the number of gestational sacs divided by the number of embryos transferred per treatment arm. Additional endpoints included cycle cancellation rate; total dose of r-hFSH administered; and number of metaphase II (MII) oocytes in ICSI patients. The endpoint of ‘total pregnancy outcome failure’ was defined *post hoc* as a combination of preclinical miscarriage (biochemical pregnancy loss), early spontaneous miscarriage (any spontaneous abortion occurring between clinical and ongoing pregnancy), late spontaneous miscarriage (any spontaneous abortion that occurred after ongoing pregnancy) and ectopic pregnancy.

After analysis of primary and secondary endpoints, a *post hoc* analysis was conducted to explore the potential impact of heterogeneity due to variability in baseline clinical characteristics on live birth rates. Live birth rate was selected as it is considered the most important outcome for infertility treatment. This analysis used a baseline severity score (BSC) which was based upon the trial's POR inclusion criteria conditions. The criteria used to define BSC were (i) age ≥40 and (ii) reduced ovarian reserve using stricter cut-offs than those used in the trial, either <2 oocytes retrieved during the most recent previous ART cycle or, if no previous cycle data were available, baseline serum AMH <0.5 ng/ml (measured at screening). The BSC for a subject could take the value 0 (mild) if none of these criteria were met; 1 (moderate) if one criterion was met; or 2 (severe) if two criteria were met.

#### Safety endpoints

Safety endpoints in this study were defined as incidence and severity of ovarian hyperstimulation syndrome (OHSS), incidence of adverse events and serious adverse events, assessed in the electronic case report form system during the trial, and local tolerability based on expected injection-site reactions (as included in the label), including injection-site pain, erythema, haematoma, swelling, and/or irritation (Humaidan *et al*., 2015; Merck, 2015a,b). Treatment-emergent adverse events (TEAEs) were defined as adverse events first occurring after the start of down-regulation. OHSS was defined as either early (occurring within 9 days after oocyte retrieval) or late (occurring after Day 10 following oocyte retrieval). Any OHSS leading to hospitalization or medical intervention was defined as a serious adverse event (Zegers-Hochschild *et al*., 2009). An additional analysis was conducted to investigate the observed difference between the two groups in TEAEs during the stimulation and post-stimulation phase.

### Statistical analysis

#### Pre-specified statistical analysis

The sample size was based on the primary endpoint (total number of oocytes retrieved per participant). Assuming a difference of one retrieved oocyte between r-hFSH/r-hLH and r-hFSH (difference based on the meta-analysis by Lehert *et al*., 2014) with a common SD of 4.5, the calculated sample size was 852 randomized participants. Including an estimated 10% dropout rate, the total trial sample size to be enroled was 946 randomized participants. This assumes an overall two-sided significance level of 0.05 and 90% power to detect the stated difference between the treatment arms.

Five populations were defined for statistical analysis:

(1) The intention-to-treat (ITT) and (2) safety populations included all randomized patients who received at least one dose of study treatment. Patients were analysed based on the treatment they were randomized to in the ITT population and analysed based on the actual treatment they received in the safety population.
(3) The per-protocol (PP) population, a subset of the ITT population, included all women who did not have any major protocol deviations that were likely to impact efficacy.
(4) The embryo transfer (ET) analysis set, a subset of the ITT population, included all patients who had at least one embryo transferred 2–3 days after oocyte retrieval.
(5) The biochemical pregnancy set (defined *post hoc*) included all randomized patients who had a positive serum hCG 15–20 days after r-hCG injection.

Primary and secondary efficacy endpoints, with the exception of the implantation rate, were analysed in the ITT population. If a patient did not undergo oocyte retrieval, the number of oocytes retrieved was counted as ‘0’ in the ITT population. The primary efficacy endpoint was also analysed in the PP population. The implantation rate was analysed in the ET set. Safety endpoints were assessed in the safety population.

The primary efficacy endpoint was analysed using a Poisson regression model with terms for treatment group, country and age category (<35 versus ≥35 years). The secondary efficacy endpoints, with the exception of the implantation rate, were analysed using a logistic regression model with the same terms as in the Poisson regression model. The implantation rate was analysed using the chi-squared test. Only *P* values for the primary endpoint (number of oocytes retrieved) in the ITT population pertain to statistical significance. All other *P* values are considered nominal as they were not adjusted for multiplicity. Summary descriptive statistics were used for all quantitative variables. SAS^®^ Version 9.2 was used to conduct the statistical analyses.

#### *Post hoc* statistical analyses

An exploratory analysis was undertaken to investigate the discrepancies between the ESPART study results and those from a recent meta-analysis (Lehert *et al*., 2014). The hypothesized discrepancy between the two studies could be attributed to differences in the clinical profile of patients at baseline (Alviggi *et al*., 2016). In the meta-analysis (Lehert *et al*., 2014), definitions of POR were author-defined and therefore, heterogeneous; all studies preceded the publication of the Bologna criteria. In the ESPART study, inclusion criteria were inspired by and incorporated the ESHRE Bologna criteria, however, these criteria may include patient subgroups with different prognoses. Furthermore, the ESPART pregnancy outcomes suggested a possible benefit of r-hLH supplementation after the establishment of pregnancy (positive hCG). Therefore, the analysis investigated whether r-hLH supplementation may provide a heterogeneous benefit depending on the baseline clinical characteristics, and if r-hLH supplementation may specifically reduce the incidence of pregnancy failure.

The analysis investigating the potential impact of heterogeneity due to variability in baseline clinical characteristics on live birth rates was conducted in the ITT population. A logistic regression model was used to test the main effect of BSC, the main treatment effect, and the interaction effect between BSC and treatment. All other logistic regression models included terms for treatment group, country and age category (<35 versus ≥35 years). The total pregnancy outcome failure rate was analysed using logistic regression in the ITT population, the ET set and the biochemical pregnancy set.

## Results

**Figure 1:**
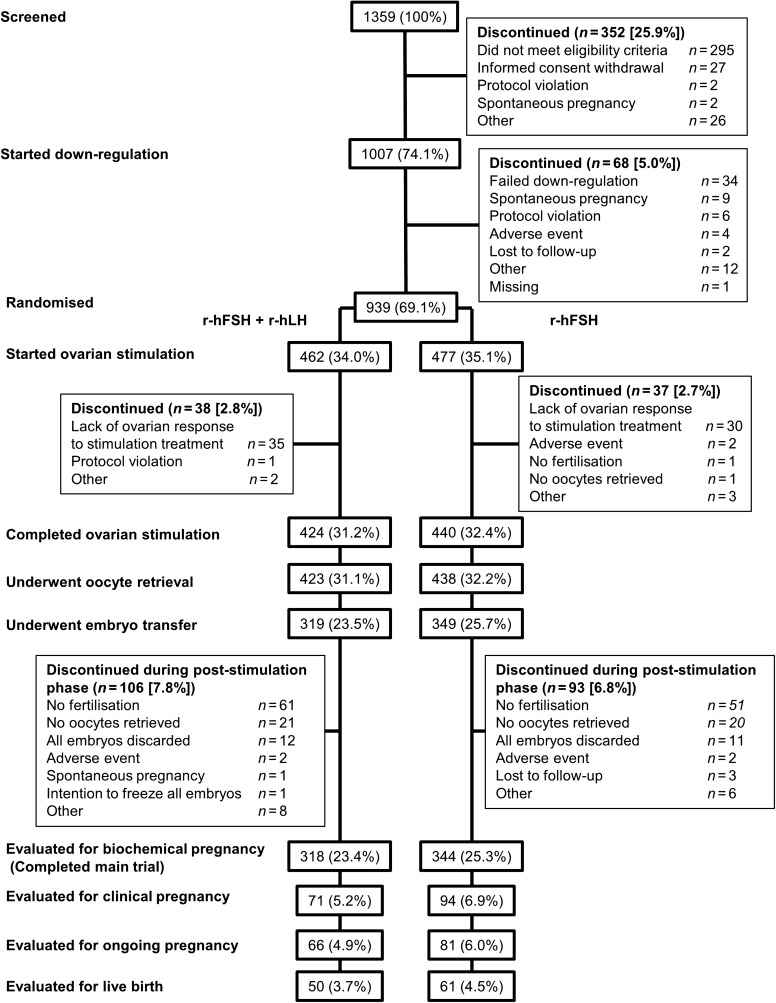
Patient disposition during the ESPART study. ESPART, Efficacy and Safety of Pergoveris in Assisted Reproductive Technology; r-hFSH, recombinant human FSH; r-hLH, recombinant human LH.

Of 1359 women screened, 1007 women were recruited and started down-regulation (Figure 1). Of these, 949 women achieved down-regulation, and 939 were randomized to undergo COS with r-hFSH/r-hLH or r-hFSH. Following randomization, 462 women started COS with r-hFSH/r-hLH and 477 started with r-hFSH. Randomization occurred at 87 sites in 15 countries. The majority of women who were randomized completed ovarian stimulation (r-hFSH/r-hLH, 91.8%; r-hFSH, 92.2%). Baseline characteristics and demographics were similar for women in the two treatment groups (Table II). Blinding compliance was maintained at all study sites.

**Table II:** Baseline demographic and clinical characteristics of patients randomized to receive either r-hFSH/r-hLH or r-hFSH monotherapy (ITT population)

	r-hFSH/r-hLH (*n* = 462)	r-hFSH (*n* = 477)	Overall (*n* = 939)
Age (years), mean (SD)	38.3 (2.9)	38.3 (3.0)	38.3 (3.0)
Race, *n* (%)			
White	439 (95.0)	454 (95.2)	893 (95.1)
Black/African American	1 (0.2)	4 (0.8)	5 (0.5)
Asian	4 (0.9)	4 (0.8)	8 (0.9)
Other	6 (1.3)	3 (0.6)	9 (1.0)
Not collected at site	12 (2.6)	12 (2.5)	24 (2.6)
BMI (kg/m^2^), mean (SD)	23.4 (3.2)	23.4 (3.1)	23.4 (3.2)
Primary infertility, *n* (%)	300 (64.9)	319 (66.9)	619 (65.9)
Duration of infertility (years), mean (SD)	4.6 (3.7)	4.4 (3.5)^*^	4.5 (3.6)^*^
Type of infertility, *n* (%)			
Female and male	186 (40.3)	185 (38.8)	371 (39.5)
Female only	276 (59.7)	292 (61.2)	568 (60.5)
Cause of female infertility, *n* (%)			
Tubal factor	68 (14.7)	85 (17.8)	153 (16.3)
Endometriosis (Grade I or II)	46 (10.0)	41 (8.6)	87 (9.3)
Ovulary dysfunction	55 (11.9)	67 (14.0)	122 (13.0)
Oligo-amenorrhoea	11 (2.4)	15 (3.1)	26 (2.8)
Primary amenorrhoea	0	0	0
Secondary amenorrhoea	1 (0.2)	1 (0.2)	2 (0.2)
Menstrual irregularity	21 (4.5)	33 (6.9)	54 (5.8)
Normal menstrual cycle with luteal defect	22 (4.8)	18 (3.8)	40 (4.3)
Other	336 (72.7)	330 (69.2)	666 (70.9)
At least one ART cycle with ≤3 oocytes retrieved	379 (82.0)	402 (84.3)	781 (83.2)
Number of previous live births, *n *(%)			
0	375 (81.2)	384 (80.5)	759 (80.8)
1	80 (17.3)	84 (17.6)	164 (17.5)
2	7 (1.5)	7 (1.5)	14 (1.5)
3	0	2 (0.4)	2 (0.2)
Antral follicle count, mean (SD)	4.9 (2.3)^†^	4.8 (2.2)^‡^	4.8 (2.2)^§^
At least one ovarian cyst with a mean size >25 mm, *n* (%)	4 (0.9)^||^	1 (0.2)^||^	5 (0.5)^‡^
AMH level (ng/ml), mean (SD)	0.58 (0.498)^¶^	0.60 (0.485)*	0.59 (0.491)^‡^
Met all three inclusion criteria for POR, *n* (%)	127 (27.5)	133 (27.9)	260 (27.7)

^*^Data missing for two patients.

^†^Data missing for seven patients.

^‡^Data missing for six patients.

^§^Data missing for 13 patients.

^||^Data missing for three patients.

^¶^Data missing for four patients.

ITT, intention-to-treat; POR, poor ovarian response; r-hFSH, recombinant human FSH; r-hLH, recombinant human LH.

### Efficacy evaluation

The mean (SD) number of oocytes retrieved in the r-hFSH/r-hLH group was 3.3 (2.71) compared with 3.6 (2.82) in the r-hFSH group (Table III). Using the Poisson regression model with terms for treatment group, country and age category (<35 versus ≥35 years), the *P*-value for the treatment effect was 0.054 without adjustment for over-dispersion (*P* = 0.182 with adjustment for over-dispersion). A similar result was obtained in the PP population (Table III). A small proportion of patients categorized as having POR through fulfilment of the inclusion criteria did not display the expected poor response, with 33 patients having at least 10 oocytes retrieved (12 patients receiving r-hFSH/r-hLH and 21 receiving r-hFSH). The baseline characteristics of these 33 patients are shown in Supplementary Table SI.

**Table III:** Primary efficacy outcome (number of oocytes retrieved) for patients randomized to receive either r-hFSH/r-hLH or r-hFSH monotherapy.

	r-hFSH/r-hLH			r-hFSH			Unadjusted		Adjusted for over-dispersion (Poisson regression model)	
	*n*	Mean (SD)	Median (range)	*n*	Mean (SD)	Median (range)	Between-group difference (95% CI)	*P*-value	Between-group difference (95% CI)	*P*-value
ITT										
Overall	462	3.3 (2.71)	3.0 (0–15)	477	3.6 (2.82)	3.0 (0–16)	–0.24 (−0.47, 0.00)	0.054	–0.24 (−0.74, 0.27)	0.182
Age <35 years	57	3.5 (2.96)	3.0 (0–12)	61	3.3 (2.50)	3.0 (0–13)	– *	0.229	– *	0.407
Age ≥35 years	405	3.3 (2.67)	3.0 (0–15)	416	3.6 (2.86)	3.0 (0–16)	–0.32 (–0.64, –0.01)	0.013	–0.32 (–0.78, 0.18)	0.085
PP										
Overall	377	3.8 (2.67)	3.0 (0–15)	395	4.0 (2.74)	3.0 (0–16)	–0.17 (–0.44, 0.09)	0.202	–0.17 (–0.72, 0.37)	0.340

*Negative of Hessian not positive definite. ITT, intention-to-treat; PP, per-protocol; r-hFSH, recombinant human FSH; r-hLH, recombinant human LH.

The predefined regression analysis of the primary endpoint included terms for country and age category (<35 versus ≥35 years), in addition to treatment group. Although neither age category nor treatment group had a significant effect on the number of oocytes retrieved, there was a significant interaction between age and treatment group (*P =* 0.042) indicating that the treatment effect was different between the two age groups. In patients aged <35 years (*n* = 118) a greater mean number of oocytes retrieved was observed with r-hFSH/r-hLH (3.5) compared with r-hFSH (3.3), whereas, in patients aged ≥35 years (*n* = 821), a lower mean number of oocytes retrieved was observed with r-hFSH/r-hLH (3.3) compared with r-hFSH (3.6). Furthermore, country was shown to have a significant effect on the number of oocytes retrieved (*P* < 0.001). However, the interaction between country and treatment group was not significant, indicating that, although the number of oocytes retrieved varied in different countries, the treatment effects were not significantly different among countries.

Secondary and additional efficacy outcome data are shown in Table IV. Clinical (14.1% versus 16.8%) and ongoing (11.0% versus 12.4%) pregnancy rates and live birth rate (10.6% versus 11.7%) were similar in both groups (r-hFSH/r-hLH and r-hFSH, respectively), despite the biochemical pregnancy rate being lower with r-hFSH/r-hLH compared with r-hFSH (17.3% versus 23.9%; *P* = 0.020). Table V shows that this corresponds to a lower incidence of total pregnancy outcome failure in patients receiving r-hFSH/r-hLH compared with those receiving r-hFSH alone, in all three relevant study populations investigated (patients receiving at least one dose of study drug [ITT population]; patients receiving an embryo transfer [ET set]; and patients having a positive pregnancy test [biochemical pregnancy set]).

**Table IV:** Secondary and other efficacy endpoints (ITT population)

	r-hFSH/r-hLH (*n* = 462)	r-hFSH (*n* = 477)	Odds ratio* (95% CI) unless otherwise indicated	*P-*value
Cancelled cycles,^†^*n* (%)	35 (7.6)	32 (6.7)	1.12 (0.68, 1.85)	0.654
Biochemical pregnancy, *n* (%)	80 (17.3)	114 (23.9)	0.68 (0.49, 0.94)	0.020
Clinical pregnancy, *n* (%)	65 (14.1)	80 (16.8)	0.83 (0.58, 1.20)	0.320
Ongoing pregnancy, *n* (%)	51 (11.0)	59 (12.4)	0.90 (0.60, 1.35)	0.599
Implantation rate, *n*/*N* (%)^‡^	79/538 (14.7)	93/597 (15.6)	0.93 (0.67, 1.29)^§^	0.675
Live birth, *n* (%)	49 (10.6)	56 (11.7)	0.91 (0.60, 1.38)	0.663
Total FSH dose administered (IU), mean (SD)	3997.7 (1188.33)^‖^	4113.6 (1193.93)	–119.3 (–269.9, 31.3)^§^	0.120
Number of MII oocytes in ICSI patients, mean (SD)	2.9 (2.07)^¶^	3.1 (2.14)**	*Unadjusted:* –0.24 (–0.64, 0.15)	*Unadjusted:* 0.063
			*Adjusted for over-dispersion:* –0.24 (–0.72, 0.23)	*Adjusted for over-dispersion:* 0.124

^*^r-hFSH/r-hLH versus r-hFSH.

^†^All cycle cancellations were due to lack of ovarian response.

^‡^*n* is the number of foetal sacs identified by transvaginal ultrasound and *N* is the total number of embryos transferred.

^§^Data are mean difference between groups (95% CI).

^**^381 patients receiving r-hFSH underwent ICSI and data were not available for 15 of these patients.

^||^Data missing for 10 patients. ^¶^360 patients receiving r-hFSH plus r-hLH underwent ICSI, and data are not available for 13 of these patients. ITT, intention-to-treat; MII, metaphase II; r-hFSH, recombinant human FSH; r-hLH, recombinant human LH.

**Table V:** Total pregnancy outcome failure*.

	r-hFSH/r-hLH	r-hFSH	Odds ratio^†^ (95% CI)	*P-*value
Total pregnancy outcome failure (ITT population), *n*/*N* (%)	31/462 (6.7)	59/477 (12.4)	0.52 (0.33, 0.82)	0.005
Total pregnancy outcome failure (ET set), *n*/*N* (%)	31/319 (9.7)	59/349 (16.9)	0.53 (0.33, 0.84)	0.007
Total pregnancy outcome failure in subjects with biochemical pregnancy, *n*/*N* (%)	31/80 (38.8)	59/114 (51.8)	0.54 (0.29, 1.01)	0.052

^*^Total pregnancy outcome failure was defined as the combination of preclinical miscarriage, clinical miscarriage (early + late) and ectopic pregnancy.

^†^r-hFSH/r-hLH versus r-hFSH.

ET set, embryo transfer analysis set; ITT, intention-to-treat; r-hFSH, recombinant human FSH; r-hLH, recombinant human LH.

In a *post hoc* analysis, the impact of BSC on the live birth rate according to treatment group was explored (Tables VI and VII). The live birth rate calculated for each BSC category and each treatment group suggested a difference of the treatment effect depending on the BSC (Table VI). In the logistic regression analysis (Table VII), in the ITT population, the parameter estimate (SE) of treatment (referent: r-hFSH) was –0.79 (0.30; *P* = 0.008) and of BSC was –1.09 (0.25; *P* < 0.001); the interaction of treatment and BSC had a parameter estimate (SE) of 1.05 (0.33; *P* = 0.001). The live birth rate in the r-hFSH group appeared to be lower in women with moderate POR (BSC = 1) and women with severe POR (BSC = 2) compared with those with mild POR (BSC = 0), whereas in the r-hFSH/r-hLH group the live birth rate was similar across all POR severity levels (Table VI).

**Table VI:** Observed (unadjusted) live birth rates according to BSC and treatment group.

BSC	Patients with a previous ART cycle	Patients with no previous ART cycle	r-hFSH/r-hLH (*N* = 462)		r-hFSH (*N* = 477)	
			*n* (%*)	Live birth rate, *n* (%^†^)	*n* (%*)	Live birth rate, *n* (%^†^)
0 (mild)	<40 years old **AND** previous ART cycle with ≥2 oocytes retrieved	<40 years old **AND** AMH > 0.5 ng/ml	170 (36.8)	18 (10.6)	156 (32.7)	34 (21.8)
1 (moderate)	≥40 years old **OR** previous ART cycle with <2 oocytes retrieved	≥40 years old **OR** AMH ≤ 0.5 ng/ml	209 (45.2)	23 (11.0)	254 (53.3)	19 (7.5)
2 (severe)	≥40 years old **AND** previous ART cycle with <2 oocytes retrieved	≥40 years old **AND** AMH ≤ 0.5 ng/ml	83 (18.0)	8 (9.6)	67 (14.0)	3 (4.5)
Overall			462 (100.0)	49 (10.6)	477 (100.0)	56 (11.7)

^*^Percentage of total population receiving each treatment.

^†^Percentage of population with BSC score for each treatment.

BSC, baseline severity score; r-hFSH, recombinant human FSH; r-hLH, recombinant human LH.

**Table VII:** Live birth rate—logistic regression model with treatment group and BSC.

Effects	ITT population		PP population	
	Parameter estimate (SE)	*P-*value	Parameter estimate (SE)	*P-*value
Intercept	–1.32 (0.19)	<0.001	–1.45 (0.22)	<0.001
Treatment	–0.79 (0.30)	0.008	–0.60 (0.33)	0.068
BSC	–1.09 (0.25)	<0.001	–0.84 (0.27)	0.002
Treatment · BSC	1.05 (0.33)	0.001	0.90 (0.35)	0.009

Reference BSC: BSC = 0 (mild); reference treatment: r-hFSH; ITT, intention-to-treat; PP, per-protocol.

### Safety evaluation

One patient who received r-hFSH/r-hLH experienced one event of mild early OHSS (Supplementary Data), and no other OHSS events were reported.

The overall incidence of TEAEs was generally similar between the two treatment groups, occurring in 25.8% and 33.3% of women treated with r-hFSH/r-hLH and r-hFSH, respectively (Table VIII). The majority of the TEAEs occurred during the stimulation and post-stimulation phases, with a low number of these assessed by the investigator as being related to either r-hFSH/r-hLH or r-hFSH (occurring in 4.8% and 4.2% of women, respectively). Most TEAEs were mild or moderate in severity in both treatment groups. Two women (0.4%) discontinued treatment with r-hFSH/r-hLH because of TEAEs compared with four (0.8%) patients who discontinued r-hFSH. The TEAEs leading to discontinuation of r-hFSH/r-hLH were ovarian rupture (post-oocyte retrieval ovarian bleeding requiring laparoscopy) and endometrial polyps. The TEAEs leading to discontinuation of r-hFSH were overdose (asymptomatic), herniated cervical vertebral disc, ovarian cyst and ovarian polyp.

**Table VIII:** Overview of adverse events (safety population).

	r-hFSH/ r-hLH (*n* = 462)	r-hFSH (*n* = 477)	Odds ratio (95% CI)
AE prior to down-regulation	38 (8.2)	36 (7.5)	1.12 (0.69, 1.82)
TEAE	119 (25.8)	159 (33.3)	0.67 (0.49, 0.91)
TEAE during down-regulation phase	59 (12.8)	75 (15.7)	0.76 (0.51, 1.14)
TEAE during stimulation and post-stimulation phase	92 (19.9)	128 (26.8)	0.67 (0.48, 0.92)
TEAE related to study drug during stimulation and post-stimulation phase	23 (5.0)	20 (4.2)	1.28 (0.68, 2.44)
Serious TEAE^*^	8 (1.7)	17 (3.6)	0.46 (0.19, 1.09)
TEAE resulting in withdrawal of study drug^†^	0	2 (0.4)	
TEAE leading to trial discontinuation^†^	2 (0.4)	4 (0.8)	

^*^All serious TEAEs occurred during the stimulation and post-stimulation phases.

^†^Subject counts too small for meaningful OR calculation.

All data are presented as *n* (%). AE, adverse event; TEAE, treatment-emergent adverse event; r-hFSH, recombinant human FSH; r-hLH, recombinant human LH.

An observed lower proportion of patients receiving r-hFSH/r-hLH compared with those receiving r-hFSH experienced TEAEs during the stimulation and post-stimulation phases (*n* = 92 [19.9%] versus *n* = 128 [26.8%]). A large proportion of these TEAEs were mild or moderate in intensity (Table IX).

**Table IX:** Overview of the proportion of patients experiencing TEAEs during the stimulation and post-stimulation phases (safety population).

	r-hFSH/r-hLH (*n* = 462)	r-hFSH (*n* = 477)
Nervous system disorders	36 (7.8)	38 (8.0)
Gastrointestinal disorders	29 (6.3)	35 (7.3)
Pregnancy, puerperium and perinatal conditions	17 (3.7)	32 (6.7)
Reproductive system and breast disorders	13 (2.8)	20 (4.2)
Maximum severity of TEAE experienced^*^		
No TEAE	370 (80.1)	349 (73.2)
Mild	64 (13.9)	88 (18.4)
Moderate	21 (4.8)	27 (5.7)
Severe	7 (1.5)	13 (2.7)

^*^Maximum severity of TEAEs experienced by each patient (no TEAE, mild, moderate and severe; this includes all TEAEs and not just those in the four System Organ Classes detailed in the table).

Data are presented as *n* (%). The four System Organ Classes most frequently associated with TEAEs during the stimulation and post-stimulation phases are presented. Reported values are for patients having experienced at least one TEAE during the stimulation and post-stimulation phases r-hFSH, recombinant human FSH; r-hLH, recombinant human LH; TEAE, treatment-emergent adverse event..

The most common TEAEs during the stimulation and post-stimulation phases, reported in >2% of either treatment group in the safety population, were headache (6.1% and 5.9% in the r-hFSH/r-hLH group and r-hFSH group, respectively), abdominal pain (2.4% and 2.5%), nausea (2.6% and 2.1%), spontaneous abortion (1.5% and 2.7%) and hot flush (2.2% and 0.8%). TEAEs of spontaneous abortion, abortion and missed abortion were reported for 13 patients treated with r-hFSH/r-hLH and 21 treated with r-hFSH. There were few serious TEAEs and the incidence was comparable between groups (1.7% and 3.6% with r-hFSH/r-hLH and r-hFSH, respectively). Serious TEAEs that occurred in more than one woman were missed abortion, which occurred in one woman treated with r-hFSH/r-hLH and three treated with r-hFSH, and abortion, which occurred in two women treated with r-hFSH. Ectopic pregnancy was reported for one patient receiving r-hFSH. There was no consistent pattern of TEAEs associated with either treatment.

## Discussion

The ESPART study is the first adequately powered, randomized controlled trial to investigate reproductive outcomes in women with POR undergoing IVF/ICSI, using selection criteria that incorporate the Bologna criteria. With 939 subjects, it is the largest study ever performed in this category of patients. Indeed, it has previously been suggested that it might be unrealistic to expect randomized controlled trials investigating POR to achieve large sample sizes (Papathanasiou, 2014). There was no statistically significant difference in the number of oocytes retrieved following COS (primary outcome) between patients receiving r-hFSH/r-hLH and those receiving r-hFSH alone and, as expected, a low average number of oocytes was retrieved in both groups. This result is contrasted by recent data from a meta-analysis in patients with POR, in which significantly more oocytes were retrieved with r-hFSH/r-hLH versus r-hFSH alone (Lehert *et al*., 2014). However, this meta-analysis did not include patients with POR classified according to the Bologna criteria, instead including studies with heterogeneous definitions of POR (Lehert *et al*., 2014).

The ESPART study did not include the complete ESHRE Bologna criteria, rather a stricter interpretation was applied to remove diagnostic subjectivity, reduce patient heterogeneity, and exclude patients with the worst reproductive prognosis. It included the objective parameters of age, an AMH level of 0.12–1.3 ng/ml and a previous ART cycle with ≤3 oocytes retrieved with a conventional stimulation protocol. Furthermore, the study randomized 939 patients stratified by age (<35 or ≥35 years) and study site to ensure adequate statistical power to detect a difference between treatment groups for the primary endpoint, the number of oocytes retrieved, and provide uniform baseline characteristics between the groups.

In the present study, the live birth rates were similar in both treatment groups (10.6% and 11.7% with r-hFSH/r-hLH and r-hFSH, respectively); interestingly, these rates were considerably higher than the live birth rates (5.5–7.4%) reported in retrospective analyses of Bologna poor ovarian responders (Polyzos *et al*., 2014; La Marca *et al*., 2015), and even lower live birth rates (2.6% per cycle) when only patients with POR undergoing natural cycle IVF were analysed retrospectively (Polyzos *et al*., 2012). This difference in live birth rate might be attributed to the ESPART study design as a randomized controlled trial with predefined inclusion criteria. Furthermore, it may reflect differences in the COS protocol used in ESPART compared with the studies included in the retrospective analyses.

Although biochemical pregnancy rates differed between treatment groups, this difference diminished over the course of the pregnancy, with similar live birth rates in both groups. A *post hoc* analysis to investigate this difference indicated that the incidence of total pregnancy outcome failure (combination of preclinical miscarriage, early or late spontaneous miscarriage and ectopic pregnancy) was lower in the r-hFSH/r-hLH group than in the r-hFSH group. This observation may relate to the potential beneficial effects of r-hLH supplementation on oocyte ‘quality’ and/or the capacity of r-hLH to support the pregnancy-sustaining function of the endometrium (Tesarik *et al*., 2003; Bosch *et al*., 2011). However, these findings should be confirmed in additional studies.

LH supplementation may reduce the rate of granulosa/cumulus cell apoptosis (a marker of oocyte quality that is positively correlated with female age) *via* increased epiregulin and amphiregulin up-regulation (Lee *et al*., 2001; Bencomo *et al*., 2006; Ruvolo *et al*., 2007; Hill and Propst, 2012). LH receptors are present in the endometrium during the window of implantation, playing an important role in embryo–endometrium cross-talk during implantation (Banerjee and Fazleabas, 2010, 2011; Hill and Propst, 2012). Moreover, other indirect/direct effects of LH on endometrial function are poorly explored and require more research. The proposed positive effects of LH supplementation on both oocyte quality and endometrial receptivity might also explain significantly increased implantation rates previously observed in patients aged ≥35 years who received r-hLH supplementation to r-hFSH for COS (Humaidan *et al*., 2004; Marrs *et al*., 2004; Barrenetxea *et al*., 2008; Matorras *et al*., 2009; Bosch *et al*., 2011; Hill *et al*., 2012).

Utilization of the Bologna POR criteria leads to a binary decision, (i.e. yes or no) as to whether or not a patient is a poor ovarian responder. However, it may be more useful to quantify the range of POR using ovarian response or reserve as an assessment tool. To this end, a *post hoc* exploratory analysis based on the key POR inclusion criteria (age, AMH and previous ART outcome; the BSC analysis) permitted a more detailed investigation of the possible effect of treatment within three distinct subgroups defined according to baseline thresholds. Interestingly, using this concept of BSC and specifically considering live birth, outcomes differed between the treatment groups; r-hFSH/r-hLH showed more promise for the patients with moderate POR (BSC = 1) or severe POR (BSC = 2), and r-hFSH was associated with better outcomes for patients with mild POR (BSC = 0). The mild POR subgroup, representing one-third of the ESPART study population, included a heterogeneous group of patients who met the ESPART inclusion criteria but were younger than 40 years old, with either three or more oocytes in a previous ART cycle or an AMH level >0.5 ng/ml in absence of a previous ART cycle. The analysis showed an interaction between BSC and treatment. However, these data need to be confirmed in additional studies stratifying patients with POR according to age, serum AMH and number of oocytes retrieved in previous ART cycles.

In conclusion, the ESPART study represents pioneering work in POR and is the largest randomized, controlled study ever performed in women with POR. The study did not meet its primary endpoint of superiority of r-hFSH/r-hLH to r-hFSH in terms of number of oocytes retrieved following COS. Furthermore, the live birth rates per cycle were similar in both groups, but considerably higher than previously reported in retrospective studies that included Bologna POR patients, suggesting that recombinant gonadotropin stimulation protocols represent an effective treatment strategy in this challenging patient category. In addition, *post hoc* analyses indicated that the incidence of total pregnancy outcome failure was significantly lower in the r-hFSH/r-hLH group than in the r-hFSH group and that live birth rate was associated with both treatment type (r-hFSH/r-hLH or r-hFSH) and POR baseline characteristics. Collectively, these data suggest that r-hFSH/r-hLH treatment may have added clinical value compared with r-hFSH alone in a subset of women with POR, but additional studies are needed to validate and confirm these observations.

## Supplementary data

Supplementary data are available at *Human Reproduction* online.

## Supplementary Material

Supplementary Table 1

Supplementary Data
