# Supplementary material for: Efficacy and safety of follitropin alfa/lutropin alfa in ART: a randomized controlled trial in poor ovarian responders
Source: Hum Reprod. 2017 Jan 28;32(3):544–55. doi: 10.1093/humrep/dew360 (PMC5850777; doi:10.1093/humrep/dew360)
Supplement: Supplementary Table 1 [file dew360suppl_table1.pdf]

Supplementary Table SI **Baseline demographics and characteristics of patients with at least 10 oocytes retrieved (ITT population).**

No. of oocytes retrieved	Age (years)	Duration of infertility (years)	AMH level (ng/ml)	AFC	No. of previous ART cycles	No. of oocytes retrieved in previous ART cycles (range)	No. of oocytes retrieved in most recent ART cycle	No. of previous live births
r-hFSH/r-hLH								
10	34	7.6	0.13	10	1	3	3	1
13	41	1.0	0.17	6	N/A	N/A	N/A	1
15	40	4.5	0.15	8	1	9	9	0
11	41	3.0	0.19	9	3	2–13	2	1
12	40	8.2	0.14	11	1	12	12	0
12	31	1.2	0.13	8	2	3–7	7	1
14	40	3.0	0.34	0	1	1	1	0
13	40	4.5	0.71	9	1	2	2	0
11	32	1.0	0.10	9	1	2	2	0
10	38	4.0	0.15	7	1	1	1	0
11	40	3.0	0.13	8	1	3	3	0
10	34	1.2	N/A	3	1	1	1	0
r-hFSH								
14	41	2.2	0.36	7	2	2–4	4	0
12	41	4.7	0.50	6	2	2–9	9	0
10	39	3.0	0.11	7	2	3–4	3	1
13	40	2.0	0.13	12	2	3–7	3	0
10	36	10.0	0.12	2	2	1–2	2	0
13	40	2.0	0.15	4	2	1–3	1	0
11	40	2.3	0.07	7	1	7	7	0
11	40	3.8	0.27	6	1	0	0	0
11	41	2.0	0.23	8	1	3	3	0
13	30	4.0	0.09	3	4	3	3	0
12	40	8.1	0.29	10	2	3	3	1
12	41	15.1	0.25	14	3	3	3	0
16	41	6.9	0.16	10	1	3	3	2
11	41	4.0	0.38	4	1	3	3	2
10	41	2.2	0.06	9	2	5–8	5	0
10	41	2.0	0.09	8	3	3–12	4	0
13	40	2.3	0.07	5	3	0–8	8	0
11	41	2.0	0.19	4	N/A	N/A	N/A	0
10	41	0.4	0.11	5	1	6	6	0
10	40	4.0	0.18	8	2	1–4	1	0
10	31	9.1	0.10	3	1	3	3	0

AFC, antral follicle count; AMH, anti-Müllerian hormone; ITT, intention-to-treat; N/A, not available; No., number; r-hFSH, recombinant human FSH; r-hLH, recombinant human LSH.
