# Supplementary material for: Efficacy and safety of follitropin alfa/lutropin alfa in ART: a randomized controlled trial in poor ovarian responders
Source: Hum Reprod. 2017 Jan 28;32(3):544–55. doi: 10.1093/humrep/dew360 (PMC5850777; doi:10.1093/humrep/dew360)
Supplement: Supplementary Data [file dew360suppl_data.pdf]

Human Reproduction, 2, 2017
doi:10.1093/humrep/dew360

human reproduction

SUPPLEMENTARY DATA

Early mild ovarian hyperstimulation syndrome (OHSS) was defined as OHSS with an onset occurring during stimulation and up to 9 days after oocyte retrieval with abdominal bloating, mild abdominal pain and enlarged ovarian size. The OHSS event occurred on the day following oocyte retrieval (16 days after the first administration of recombinant human FSH (r-hFSH)/recombinant human LH (r-hLH) and 5 days after the last administration) and resolved after 4 days; seven oocytes were retrieved from this patient. At baseline, the patient was 41 years old (40 years old at the time of eligibility and informed consent), and had a BMI of 27.54 kg/m^2, an anti-Müllerian hormone level of 0.07 ng/ml and an antral follicle count of 4. The patient had a 4-year duration of infertility and had four previous ART cycles (3–8 oocytes retrieved; with 3 retrieved in the most recent cycle) with no previous live births.
